# Investigation of peripheral inflammatory biomarkers in association with suicide risk in major depressive disorder

**DOI:** 10.3389/fpsyt.2024.1321354

**Published:** 2024-01-29

**Authors:** Borbála Pethő, Márton Áron Kovács, Diána Simon, Tünde Tóth, András Sándor Hajnal, Tímea Csulak, Dóra Hebling, Noémi Albert, Eszter Varga, Márton Herold, Péter Osváth, Viktor Vörös, Tamás Tényi, Róbert Herold

**Affiliations:** ^1^ Department of Psychiatry and Psychotherapy, Clinical Center, Medical School, University of Pécs, Pécs, Hungary; ^2^ Department of Immunology and Biotechnology, Clinical Center, Medical School, University of Pécs, Pécs, Hungary; ^3^ Department of Anatomy, Clinical Center, Medical School, University of Pécs, Pécs, Hungary; ^4^ Department of Pediatrics, Clinical Center, Medical School, University of Pécs, Pécs, Hungary

**Keywords:** major depressive disorder, suicidality, suicide risk, neutrophil-to-lymphoycte ratio, monocyte-to-lymphocyte ratio, laboratory parameters, biomarker, inflammation

## Abstract

Suicide is the most severe complication of major depressive disorder (MDD). Novel research assumes the role of immunological dysregulation in the background – several studies have reported alterations in the number of inflammatory cells related to both MDD and suicidality. There are currently no objective, routinely measured parameters to indicate suicidal vulnerability. However, altered inflammatory cell numbers and ratios have been proposed as potential biomarkers of suicide risk (SR). The present research aims to examine changes of these values related to increased SR in MDD as an assumed inflammatory state. We investigated laboratory parameters of psychiatric in-patients diagnosed with MDD (*n* = 101) retrospectively. Individuals with recent suicide attempt (SA) (*n* = 22) and with past SA (*n* = 19) represented the high SR group. MDD patients with no history of SA (*n* = 60) composed the intermediate SR group. We compared the number of neutrophil granulocytes, monocytes, lymphocytes, platelets, white blood cell count (WBC), neutrophil-to-lymphocyte (NLR), monocyte-to-lymphocyte (MLR), platelet-to-lymphocyte ratio (PLR), mean platelet volume (MPV), red blood cell distribution width (RDW) and erythrocyte sedimentation rate (ESR). Furthermore, we evaluated alterations of these parameters related to antidepressant (AD) and antipsychotic (AP) treatment, which have been proved to have anti-inflammatory effects. We found a significant increase in neutrophil granulocyte count, NLR, monocyte count, MLR, WBC and ESR in patients with recent SA compared to patients with no history of SA. Moreover, there was a significant elevation in monocyte count, MLR, ESR and RDW in patients with high SR compared to patients with intermediate SR. AD treatment resulted in a significant decrease in neutrophil granulocyte count and NLR, however, it did not affect monocyte count and MLR. Assuming immunological mechanisms in the background of MDD and suicidality, our findings support the role of NLR as a biomarker of acute SR, though its alterations may be masked by possible anti-inflammatory effects of AD treatment in the long term. However, MLR, a marker exhibiting changes which are not attenuated by pharmacotherapy, may be a possible indicator of both acute and long-term suicidal vulnerability.

## 1 Introduction

To this day, suicide is a global issue of increasing importance. Taking one life every 40 seconds (1), it accounts for an estimated 800 thousand deaths every year and at least twenty times as many attempts are made (2). Although the latter is more common in women, men are three times more likely to complete suicide, according to the gender paradox (3). Suicidality includes suicidal ideations, plans and attempts (4), all of which place a burden on both the direct environment and society as a whole. Assessing the risk remains a great challenge for clinicians: according to research, 30% of patients committing suicide have met with their physician in the month prior to the event (5) and up to 80% of individuals taking their own life failed to disclose their intention the last time they contacted their doctor (6). Currently, the risk stratification is partly based on self-report (6) and its efficacy might be diminished by the lack of trust and cooperation on behalf of a patient in an acute state of a psychiatric illness. There is a great demand for objective, easily accessible and reproducible, cost-effective laboratory parameters which could be measured as part of a routine examination to indicate suicidal vulnerability and thus, as a supplement to pre-existing methods, help evaluate suicide risk (SR) more precisely.

As described in a study, 8.6% of individuals hospitalized for suicidality will eventually die by taking their own lives (7). Apart from the history of a previous attempt (6), the risk factors of suicide may be distal, also known as predisposing factors, which indirectly increase susceptibility. These include certain personality traits (e.g. impulsive, aggressive), genetic characteristics and adverse early life experiences (8). While distal factors determine suicidal vulnerability from the outset, proximal or precipitating factors directly result in suicidality. To the latter group belong among others stress, alcohol or drug abuse and psychiatric disorders (8). Major depressive disorder (MDD) not only contributes greatly to the global burden of diseases (9) but remains one of the greatest risk factors for suicidality (10): almost 15% of MDD patients will commit suicide (11).

Recent research assumes the altered function of the immune system in association with MDD and suicidality: elevated levels of pro-inflammatory cytokines – low molecular weight regulator glycoproteins secreted by various cells, including leukocytes and platelets – have been detected in patients diagnosed with MDD and individuals having attempted suicide compared to healthy and non-suicidal controls (12, 13). Although the exact etiology is yet to be discovered, several studies have correlated changes in the number of cells secreting these mediators with the development of MDD (14) and the emergence of suicidality (15). Therefore, alterations of neutrophil granulocyte, monocyte, platelet, lymphocyte and white blood cell count (WBC) as well as neutrophil-to-lymphocyte (NLR), monocyte-to-lymphocyte (MLR) and platelet-to-lymphocyte ratios (PLR) have been proposed as potential biomarkers of SR (16).

Studies investigating the efficiency of NLR as a biomarker of suicidal vulnerability have provided the most promising results. A significant increase in NLR has been described in suicide attempters (17). Moreover, Velasco et al. have found NLR to be significantly associated with suicidality in MDD patients (15).

In most cases, attempters are psychiatric patients undergoing antidepressant (AD) or antipsychotic (AP) medication. These types of pharmacotherapies have exhibited anti-inflammatory effects (18, 19), which further complicates the relationship between immunological markers and suicidal vulnerability in MDD.

In the current research, we investigated alterations of immune cell numbers and ratios in MDD patients in relation to SR, taking into account the effects of AD and AP medication on these values. We aimed to contribute to a better understanding of the immunological changes related to suicidality in MDD, in the hope that in the future, these parameters may be used as biomarkers to indicate suicidal vulnerability and thus closer monitoring, longer duration of hospitalization, pharmacotherapy and psychotherapy for patients with high SR may help prevent more suicide-related deaths.

## 2 Materials and methods

We collected data retrospectively from psychiatric patients (*n* = 101) diagnosed with MDD according to the Diagnostic and Statistical Manual of Mental Disorders, Fifth Edition (DSM-5), receiving inpatient care and pharmacotherapy at the Department of Psychiatry and Psychotherapy, Medical School, Clinical Center, University of Pécs. Data collection was carried out between January 2015 and December 2020. We investigated the number of neutrophil granulocytes, monocytes, lymphocytes, platelets and WBC. We measured NLR, MLR, PLR, mean platelet volume (MPV), erythrocyte sedimentation rate (ESR) and red blood cell distribution width (RDW). Three patient groups were formed based on the presence of a suicide attempt (SA): recent (≤ 48 hours prior), past (> 48 hours prior) attempters and patients with no history of SA. Further dividing individuals according to SR, participants with recent or past SA represented the high SR group and MDD patients with no history of SA were considered as individuals with intermediate SR. Recent SA was carried out by self-poisoning with benzodiazepines. The participants were of Caucasian ethnicity, with age ranging from 26 to 79 years old. Further sociodemographic and clinical characteristics of the patient groups are summarized in **Table 1**.

**Table 1:** Sociodemographic and clinical characteristics of the patient groups.

	Total sample *(n* = 101)	Recent SA *(n* = 19)	Past SA *(n* = 22)	No history of SA *(n* = 60)	Significance (*p*)	High SR *(n* = 41)	Intermediate SR *(n* = 60)	Significance (*p*)
Males (*n* [%])	26 (25,74)	4 (21,05)	6 (27,27)	16 (26,67)	*p* > 0.05	10 (24,39)	16 (26,67)	0.822
Females (*n* [%])	75 (74,26)	15 (78,95)	16 (72,73)	44 (73,33)		31 (75,61)	44 (73,33)	
Age (mean ± SD)	54,29 ± 1,15	56,89 ± 2,98	54,00 ± 2,06	53,57 ± 1,52	*p* > 0.05	55,34 ± 1,76	53,57 ± 1,52	0.450
Smoking (*n* [%])	37 (36,63)	6 (31,58)	12 (54,55)	19 (31,67)	*p* > 0.05	18 (43,90)	19 (31,67)	0.293
Concomitant diabetes mellitus (*n* [%])	12 (11,88)	4 (21,05)	2 (9,09)	6 (10,00)	*p* > 0.05	6 (14,63)	6 (10,00)	*p* > 0.05
Concomitant hypertension (*n* [%])	31 (30,69)	6 (31,58)	6 (27,27)	19 (31,67)	*p* > 0.05	12 (29,27)	19 (31,67)	*p* > 0.05
Positive family history of psychiatric illness (*n* [%])	52 (51,49)	14 (63,63)	8 (42,11)	30 (50,00)	*p* > 0.05	22 (42,31)	30 (50,00)	0.840
**AP treatment (*n* [%])**	69 (68,32)	8 (42,11)	20 (90,91)	41 (68,33)	***p_past SA vs recent SA_*** ≤ **0.01** ***p_past SA vs no history of SA_ =* 0.047** *p_recent SA vs no history of SA_* > 0.05	28 (68,29)	41 (68,33)	*p* > 0.999

For statistical analysis, the chi-squared test with Fisher’s exact test, unpaired t-test and one-way ANOVA with Tukey’s multiple comparison test were used. AP, antipsychotic; SA, suicide attempt; SD, standard deviation; SR, suicide risk. Bold values denote statistical significance.

The exclusion criteria were acute or chronic inflammatory illnesses, autoimmune diseases, hematological or oncological disorders and current treatment with anti-inflammatory or immunosuppressive medication. Individuals having attempted suicide by causing physical injury were excluded from the recent SA group. Patients exhibiting psychotic symptoms were not included. None of the participants had a history of ECT treatment.

There were no significant differences regarding age, gender, smoking or the presence of a concomitant medical condition (hypertension, diabetes mellitus) between the patient groups. All of the participants were undergoing pharmacological AD treatment and a subset of them received AP therapy. AD treatment consisted of selective serotonin reuptake inhibitor (SSRI), serotonin noradrenaline reuptake inhibitor (SNRI), noradrenaline and specific serotonergic antidepressant (NaSSA), serotonin antagonist and reuptake inhibitor (SARI) and norepinephrine-dopamine reuptake inhibitor (NDRI) medication. AP treatment included first, second and third generation agents. In the past SA group, significantly more patients were treated with AP medication compared to the recent SA and no history of SA groups, therefore we paid close attention to the alterations of the investigated values related to AP treatment.

Laboratory tests were performed at the Department of Laboratory Medicine, Medical School, Clinical Center, University of Pécs. For the statistical analysis, GraphPad Prism version 9.5.0 Windows program (GraphPad Software, San Diego, CA, USA, www.graphpad.com, accessed in 2022) and MedCalc 16.8 Windows program (MedCalc Software Ltd., Ostend, Belgium, www.medcalc.org, accessed in 2023) were used. Statistical operations were carried out first by performing a descriptive analysis and then by determining the distribution of samples. Outliers were identified using the robust regression and outlier removal (ROUT) method and excluded from further statistical analyses. For comparisons between groups, the unpaired t-test, Mann-Whitney U test, Brown-Forsythe and ordinary one-way ANOVA test, Kruskal-Wallis test and Tukey’s, Dunn’s or Dunnett’s multiple comparison analyses were used. To compare two normally distributed groups with different homogeneity of variance, Welch’s correction was applied. For testing the categorical variables, the chi-squared analysis with Fisher’s exact test was performed. The receiver operating characteristic (ROC) curve analysis was used to assess the overall diagnostic performance and to determine the cut-off value, specificity and sensitivity of laboratory parameters. In all cases, *p* ≤ 0.05 was considered statistically significant.

## 3 Results

### 3.1 Inflammatory markers in patients with recent SA and no history of SA

**Figure 1:**
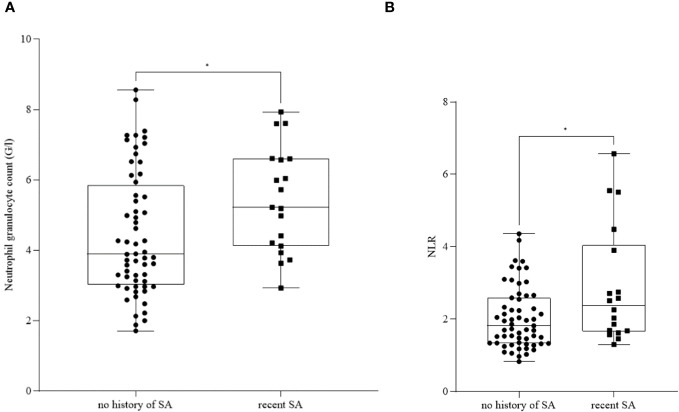
Neutrophil granulocyte count **(A)** and neutrophil-to-lymphocyte ratio (NLR) **(B)** in patients with no history of suicide attempt (SA) and recent SA. The box plot diagram represents the interquartile range and median values. Whiskers indicate the most extreme observations. The individual values are presented with black dots (patients with no history of SA, *n_neutrophil granulocyte_ =* 19*, n_NLR_ =* 19) and squares (patients with recent SA, *n_neutrophil granulocyte_* = 60, *n_NLR_* = 60). For statistical analysis, the Mann-Whitney U test was used. *p ≤ 0.05.

Neutrophil granulocyte count (**Figure 1A**) and NLR (**Figure 1B**) were significantly elevated in patients with recent SA (*n_neutrophil granulocyte_ =* 19, mean: 5.42 ± 1.49; *n_NLR_ =* 19, mean: 3.26 ± 2.24) compared to patients with no history of SA (*n_neutrophil granulocyte_ =* 60, mean: 4.44 ± 1.76; *n_NLR_ =* 60, mean: 2.10 ± 1.01) (*p_neutrophil granuloctyte_ =* 0.016; *p_NLR_ =* 0.031).

**Figure 2:**
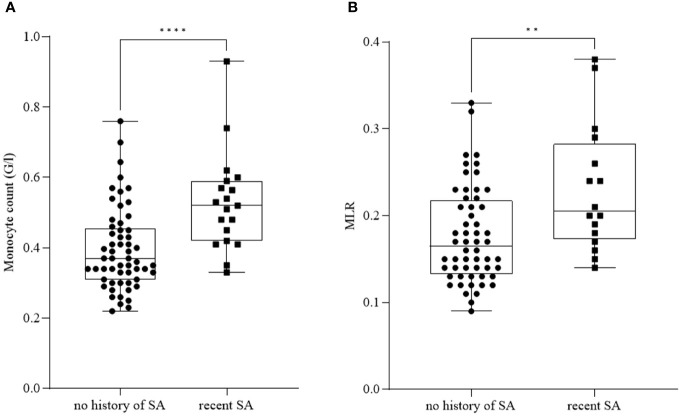
Monocyte count **(A)** and monocyte-to-lymphocyte ratio (MLR) **(B)** in patients with no history of suicide attempt (SA) and recent SA. The box plot diagram represents the interquartile range and median values. Whiskers indicate the most extreme observations. The individual values are presented with black dots (no history of SA, *n_monocyte_=* 57*, n_MLR_ =* 56) and squares (patients with recent SA, *n_monocyte_=* 19*, n_MLR_ =* 16). For statistical analysis, the Mann-Whitney U test was used. **p ≤ 0.01, ****p ≤ 0.0001.

Furthermore, we found a significant increase in monocyte count (**Figure 2A**) and MLR (**Figure 2B**) in patients with recent SA (*n_monocyte_ =* 19, mean: 0.53 ± 0.14; *n_MLR_ =* 16, mean: 0.23 ± 0.07) compared to patients with no history of SA (*n_monocyte_ =* 57, mean: 0.40 ± 0.12; *n_MLR_ =* 56, mean: 0.18 ± 0.06) (*p_monocyte_ ≤* 0.0001; *p_MLR_ =* 0.005).

Considering further inflammatory parameters (**Table 2**), ESR and WBC were significantly elevated in patients with recent SA (*n_ESR_ =* 12; *n_WBC_ =* 19) compared to patients with no history of SA (*n_ESR_ =* 43; *n_WBC_ =* 60) (*p_ESR_ =* 0.037; *p_WBC_ =* 0.048).

**Table 2:** Inflammatory parameters in patients with no history of suicide attempt (SA) and recent SA.

Laboratory parameters	Patient groups	Number of patients *(n)*	Mean ± SD	Significance *(p)*
Lymphocyte count	No history of SA	60	2.25 ± 0.65	0.274
	Recent SA	19	2.06 ± 0.72	
Platelet count	No history of SA	60	256.3 ± 69.67	0.813
	Recent SA	18	253.0 ± 44.94	
PLR	No history of SA	59	123.2 ± 47.89	0.954
	Recent SA	16	119.5 ± 33.02	
MPV	No history of SA	53	9.97 ± 1.32	0.557
	Recent SA	19	9.76 ± 1.12	
**WBC**	No history of SA	60	7.33 ± 2.18	**0.048**
	Recent SA	19	8.21 ± 1.42	
**ESR**	No history of SA	43	7.74 ± 5.07	**0.037**
	Recent SA	12	15.33 ± 11.86	
RDW	No history of SA	54	12.99 ± 0.67	0.245
	Recent SA	18	13.52 ± 1.30	

For statistical analysis, the unpaired t-test with Welch’s correction and Mann-Whitney U test were used. ESR, erythrocyte sedimentation rate; MPV, mean platelet volume; PLR, platelet-to-lymphocyte ratio; RDW, red blood cell distribution width; SD, standard deviation; WBC, white blood cell count. Bold values denote statistical significance.

### 3.2 Inflammatory markers in relation to SR

**Figure 3:**
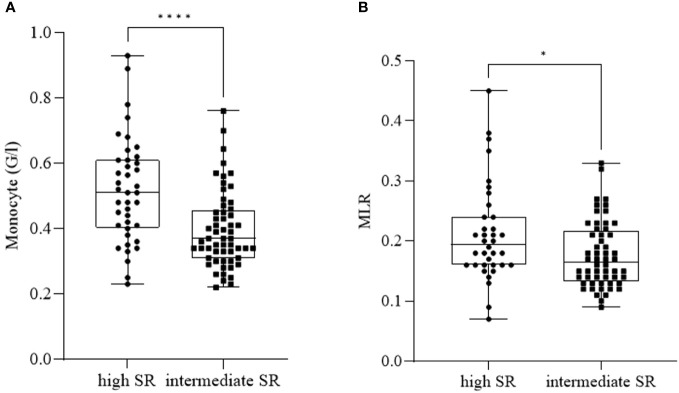
Monocyte count **(A)** and monocyte-to-lymphocyte ratio (MLR) **(B)** in patients with high suicide risk (SR) and intermediate SR. The box plot diagram represents the interquartile range and median values. Whiskers indicate the most extreme observations. The individual values are presented with black dots (patients with high SR, *n_monocyte_=* 40*, n_MLR_ =* 36) and squares (patients with intermediate SR, *n_monocyte_=* 57*, n_MLR_ =* 56). For statistical analysis, the Mann-Whitney U test was used. *p ≤ 0.05, ****p ≤ 0.0001.

We detected a significant increase in monocyte count (**Figure 3A**) and MLR (**Figure 3B**) in patients with high SR (*n_monocyte_ =* 40, mean: 0.52 ± 0.16; *n_MLR_ =* 36, mean: 0.21 ± 0.08) compared to patients with intermediate SR (*n_monocyte_ =* 57, mean: 0.40 ± 0.12; *n_MLR_ =* 56, mean: 0.18 ± 0.06) (*p_monocyte_ ≤* 0.0001; *p_MLR_ =* 0.020).

As for further inflammatory parameters (**Table 3**), ESR and RDW were significantly elevated in patients with high SR (*n_ESR_ =* 29; *n_RDW_ =* 39) compared to patients with intermediate SR (*n_ESR_ =* 43; *n_RDW_ =* 56) (*p_ESR_* = 0.041; *p_RDW_ =* 0.037).

**Table 3:** Inflammatory parameters in patients with high suicide risk (SR) and intermediate SR.

Laboratory parameters	Patient groups	Number of patients (*n*)	Mean ± SD	Significance (*p*)
Neutrophil granulocyte count	High SR	40	4.81 ± 1.61	0.203
	Intermediate SR	60	4.44 ± 1.76	
NLR	High SR	38	2.24 ± 1.30	0.776
	Intermediate SR	59	2.03 ± 0.85	
Lymphocyte count	High SR	40	2.36 ± 0.90	0.503
	Intermediate SR	60	2.25 ± 0.65	
Platelet count	High SR	40	276.5 ± 67.87	0.155
	Intermediate SR	60	256.3 ± 69.67	
PLR	High SR	35	115.1 ± 37.78	0.491
	Intermediate SR	60	122.9 ± 47.54	
MPV	High SR	39	9.89 ± 1.09	0.633
	Intermediate SR	53	9.97 ± 1.32	
WBC	High SR	40	7.90 ± 1.61	0.140
	Intermediate SR	60	7.33 ± 2.18	
**ESR**	High SR	29	14.14 ± 11.50	**0.041**
	Intermediate SR	43	7.74 ± 5.07	
**RDW**	High SR	39	13.45 ± 1.02	**0.037**
	Intermediate SR	56	13.07 ± 0.78	

For statistical analysis, the unpaired t-test with Welch’s correction and Mann-Whitney U test was used. ESR, erythrocyte sedimentation rate; MPV, mean platelet volume; NLR, neutrophil-to-lymphocyte ratio; PLR, platelet-to-lymphocyte ratio; RDW, red blood cell distribution width; SD, standard deviation; WBC, white blood cell count. Bold values denote statistical significance.

### 3.3 Effects of AD and AP therapy

As all of the participants were undergoing AD treatment, we aimed to observe the effect of pharmacotherapy on the investigated parameters. We divided data into four groups according to the received dose of AD medication: under or 100% (*n_NLR_ =* 40, mean: 1.98 ± 0.80; *n_MLR_ =* 40, mean: 0.19 ± 0.06), 200% (*n_NLR_* = 25, mean: 2.44 ± 1.33; *n_MLR_=* 23, mean: 0.20 ± 0.08), 300% (*n_NLR_ =* 22, mean: 1.97 ± 0.90; *n_MLR_ =* 20, mean: 0.17 ± 0.06), 400% or above (*n_NLR_ =* 8, mean: 1.47 ± 0.37; *n_MLR_ =* 8, mean: 0.16 ± 0.03) the minimal effective daily dose (20).

**Figure 4:**
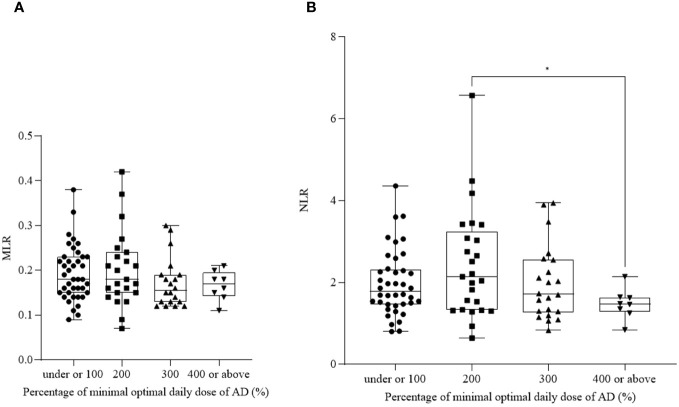
Neutrophil-to-lymphocyte ratio (NLR) **(A)** and monocyte-to-lymphocyte ratio (MLR) **(B)** in patients receiving antidepressant (AD) therapy. The box plot diagram represents the interquartile range and median values. Whiskers indicate the most extreme observations. The individual values are presented with black dots (under or 100% of minimal effective daily dose, *n_NLR_ =* 40*, n_MLR_ =* 40), squares (200% of minimal effective daily dose, *n_NLR_ =* 25*, n_MLR_ =* 23), upward triangles (300% of minimal effective daily dose, *n_NLR_ =* 22*, n_MLR_ =* 20) and downward triangles (400% or above of minimal effective daily dose, *n_NLR_ =* 8*, n_MLR_ =* 8). For statistical analysis, the Kruskal-Wallis and Brown-Forsythe with Dunn’s and Dunnett’s multiple comparison test were used. *p ≤ 0.05.

NLR (**Figure 4A**) was affected by AD therapy. We detected a significantly lower value in the group „400 or above” compared to the group „200” (*p_NLR_* = 0.016). However, MLR (**Figure 4B**) remained unaffected by AD medication, with no significant differences between the groups (*p_MLR_ =* 0.321).

As for further parameters (**Table 4**), neutrophil granulocyte count was significantly decreased in the group „400 or above” (*n =* 7, mean: 2.91 ± 0.69) compared to the group „under or 100” (*n =* 43, mean: 4.81 ± 1.71) (*p =* 0.0163). The two groups did not differ significantly regarding the rest of the values (*p* > 0.05).

**Table 4:** Inflammatory parameters in patients receiving antidepressant (AD) therapy.

Laboratory parameters	Patient groups	Number of patients (*n*)	Mean ± SD	Significance (*p*)
**Neutrophil granulocyte count**	Under or 100	43	4.81 ± 1.71	***p_200 vs 400 =_* 0.0163** *p_between other groups_* > 0.05
	200	25	4.47 ± 1.50	
	300	24	4.69 ± 1.82	
	400 or above	7	2.91 ± 0.69	
Monocyte count	Under or 100	42	0.47 ± 0.16	*p* > 0.05
	200	24	0.43 ± 0.15	
	300	24	0.45 ± 0.16	
	400 or above	8	0.39 ± 0.15	
Lymphocyte count	Under or 100	42	2.34 ± 0.67	*p* > 0.05
	200	25	2.14 ± 0.39	
	300	24	2.26 ± 0.66	
	400 or above	8	2.35 ± 0.80	
Platelet count	Under or 100	43	268.0 ± 60.50	*p* > 0.05
	200	25	263.4 ± 76.13	
	300	24	256.0 ± 77.68	
	400 or above	8	273.3 ± 27.14	
PLR	Under or 100	43	122.3 ± 46.85	*p* > 0.05
	200	25	138.2 ± 60.01	
	300	23	118.2 ± 49.93	
	400 or above	8	125.5 ± 51.62	
MPV	Under or 100	40	10.07 ± 1.14	*p* > 0.05
	200	23	9.90 ± 1.35	
	300	24	9.65 ± 1.22	
	400 or above	6	9.61 ± 2.08	
WBC	Under or 100	43	7.91 ± 2.00	*p* > 0.05
	200	25	7.30 ± 1.69	
	300	24	7.58 ± 2.01	
	400 or above	8	6.39 ± 2.39	
ESR	Under or 100	29	9.59 ± 7.76	*p* > 0.05
	200	20	8.00 ± 5.19	
	300	10	14.30 ± 9.57	
	400 or above	8	14.63 ± 11.53	
RDW	Under or 100	41	13.37 ± 0.98	*p* > 0.05
	200	24	13.30 ± 0.89	
	300	23	13.19 ± 0.94	
	400 or above	8	12.73 ± 0.80	

For statistical analysis, the Kruskal-Wallis and one-way ANOVA test were used with Dunn’s and Tukey’s post-hoc analysis. ESR, erythrocyte sedimentation rate; MPV, mean platelet volume; PLR, platelet-to-lymphocyte ratio; RDW, red blood cell distribution width; SD, standard deviation; WBC, white blood cell count. Bold values denote statistical significance.

**Figure 5:**
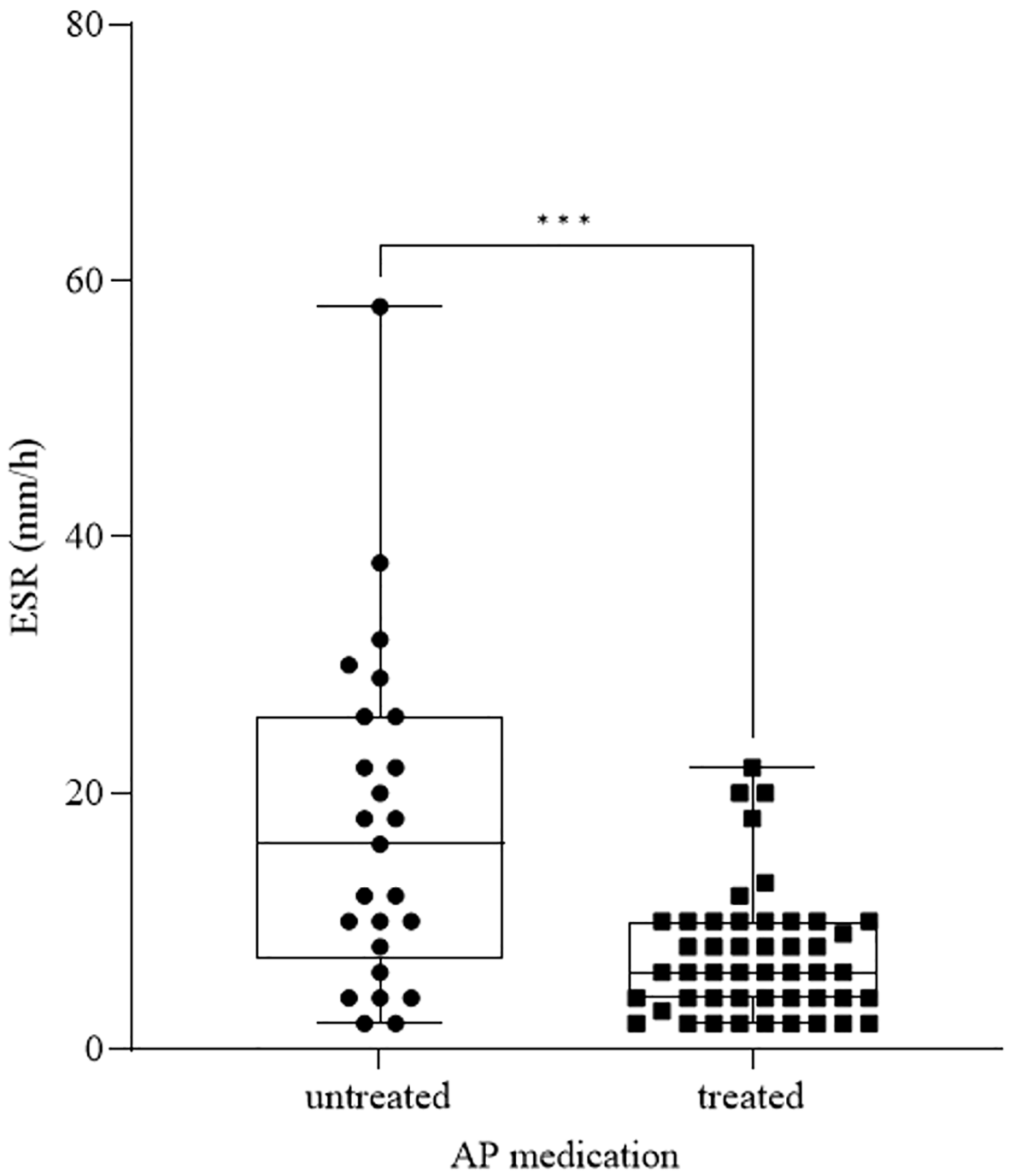
ESR in patients receiving antipsychotic (AP) therapy and untreated patients. The box plot diagram represents the interquartile range and median values. Whiskers indicate the most extreme observations. The individual values are presented with black dots (untreated, *n =* 25) and squares (treated, *n =* 48). For statistical analysis, the Mann-Whitney U test was used. ***p ≤ 0.001.

Observing the effect of AP treatment, we found a significant decrease in ESR (**Figure 5**) in patients undergoing AP medication (*n_ESR_ =* 48, mean: 7.23 ± 4.96) compared to untreated patients (*n_ESR_ =* 25, mean: 17.56 ± 13.28) (*p_ESR_ =* 0.0002). There were no significant differences between the recent SA and no history of SA groups regarding the number of patients receiving AP medication. Although there were more AP treated patients in the high SR group compared to the intermediate SR group, we detected significantly higher ESR values in the high SR group.

The rest of the investigated parameters (**Table 5**) were not significantly affected by AP medication (*p >* 0.05).

**Table 5:** Inflammatory parameters in patients receiving antipsychotic (AP) therapy and untreated patients.

Laboratory parameters	Patient groups	Number of patients (*n*)	Mean ± SD	Significance (*p*)
Neutrophil granulocyte count	Untreated	33	4.85 ± 1.58	0.294
	Treated	67	4.46 ± 1.76	
NLR	Untreated	29	2.00 ± 0.75	0.596
	Treated	64	1.97 ± 0.89	
Monocyte count	Untreated	32	0.45 ± 0.14	0.802
	Treated	66	0.45 ± 0.17	
MLR	Untreated	30	0.19 ± 0.07	0.716
	Treated	61	0.18 ± 0.06	
Lymphocyte count	Untreated	33	2.24 ± 0.67	0.848
	Treated	67	2.32 ± 0.80	
Platelet count	Untreated	33	259.8 ± 67.51	0.643
	Treated	67	266.7 ± 70.59	
PLR	Untreated	31	115.9 ± 31.83	0.686
	Treated	67	128.2 ± 56.08	
MPV	Untreated	29	9.71 ± 1.41	0.369
	Treated	64	9.97 ± 1.21	
WBC	Untreated	33	7.78 ± 1.77	0.436
	Treated	67	7.45 ± 2.08	
RDW	Untreated	31	13.15 ± 0.96	0.444
	Treated	66	13.34 ± 1.01	

For statistical analysis, the Mann-Whitney U test and unpaired t-test with Welch’s correction were used. ESR, erythrocyte sedimentation rate; MLR, monocyte-to-lymphocyte ratio; MPV, mean platelet volume; NLR, neutrophil-to-lymphocyte ratio; PLR, platelet-to-lymphocyte ratio; RDW, red blood cell distribution width; SD, standard deviation; WBC, white blood cell count.

### 3.4 Investigation of the diagnostic value of inflammatory parameters

**Figure 6:**
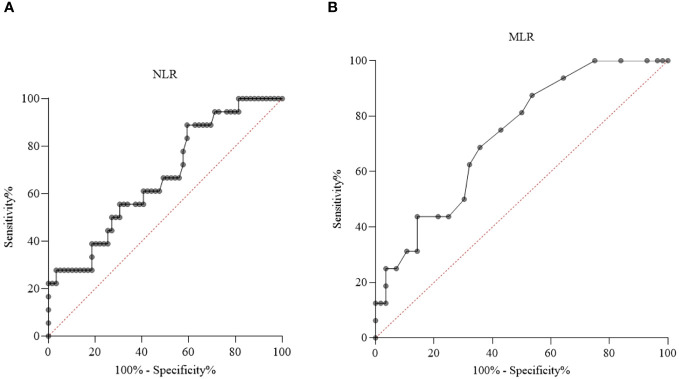
Receiver operating characteristic (ROC) curve analysis of neutrophil-to-lymphocyte ratio (NLR) **(A)** and monocyte-to-lymphocyte ratio (MLR) **(B)** in patients with recent suicide attempt (SA) and no history of SA.

We examined the diagnostic value of NLR (**Figure 6A**) and MLR (**Figure 6B**) in differentiating patients with recent SA (*n_NLR_* = 1*9*; *n_MLR_* = 16) from individuals with no history of SA (*n_NLR_ =* 60; *n_MLR_ =* 56). According to the AUC value (AUC_NLR_: 0.669, 95% CI_NLR_ = 0.529 – 0.809, *p_NLR_ =* 0.031; AUC_MLR_: 0.728, 95% CI_MLR_ = 0.60 – 0.857, *p_MLR_ =* 0.006), the diagnostic performance of NLR was limited. The diagnostic performance of MLR was acceptable. Calculated according to the Youden-index, the optimal cut-off value was 1.54 for NLR (sensitivity = 88.2%, specificity = 40.7%) and 0.14 for MLR (sensitivity = 87.5%, specificity = 46.4%) in predicting SA.

As for further inflammatory parameters (**Table 6**), monocyte count had an acceptable diagnostic performance. Neutrophil granulocyte count, WBC and ESR had a limited diagnostic value.

**Table 6:** Diagnostic value of inflammatory parameters exhibiting a significant difference between patients with no history of suicide attempt (SA) and recent SA according to the receiver operating characteristic (ROC) curve.

Laboratory parameters	Patient groups	Number of patients (*n*)	Significance (*p*)	AUC	95% CI	Cut-off value	Specificity (%)	Sensitivity (%)
**Neutrophil granulocyte count**	No history of SA	60	**0.017**	0.683	0.560 – 0.806	3.62 <	41.7	94.7
	Recent SA	19						
**Monocyte count**	No history of SA	57	**0.0002**	**0.790**	0.681 – 0.897	0.40 <	61.4	89.5
	Recent SA	19						
**WBC**	No history of SA	60	**0.042**	0.656	0.532 – 0.779	7.31 <	60	78.9
	Recent SA	19						
**ESR**	No history of SA	43	**0.040**	0.696	0.513 – 0.878	20 <	100	33.33
	Recent SA	12						

ESR, erythrocyte sedimentation rate; WBC, white blood cell count. Bold values denote statistical significance.

## 4 Discussion

Recent research suggests the potential role of inflammatory processes in the background of MDD and the emergence of suicidality. It has been known for decades that antibodies targeting molecular components of certain brain regions can induce structural and biochemical changes in several neuronal processes, resulting in emotional and behavioral abnormalities (21). For a long time, the blood-brain barrier was assumed to provide complete protection and independence for the central nervous system (CNS) against peripheral immunological mechanisms. Today it is well known that in certain conditions, peripherally secreted cytokines are able to pass through the blood-brain barrier due to its altered permeability and thus be present in the CNS, where, along with mediators produced locally by activated cells, they can directly affect certain neuronal networks (22) and the malfunction of the latter is assumed to play a role in the pathogenesis of MDD. Several studies have investigated the effect of these mediators on serotonin (23) and dopamine metabolism (24) via various molecular pathways (25, 26). According to the monoamine theory (27), a reduction in the level of these mediators is responsible for the development of depressive symptoms. Furthermore, cytokines alter the functioning of the hypothalamus-pituitary-adrenal axis (28), leading to changes in cognition, behavior, affect and personality, which are characteristic of MDD (29), thus supporting the role of inflammatory processes in the pathogenesis of the illness (30). In addition, the declination of memory functions and further cognitive processes is often observed in MDD and considered a consequence of cytokine expression (31, 32).

Since cytokines play a key role in carrying out immunological mechanisms, alterations in their levels may be indicative of the ongoing inflammatory processes. We have reason to assume that the pathomechanism of MDD and the development of inflammatory response are tightly bound together, with the processes mutually potentiating one another. As previously described, in a subset of patients, the activated immune response plays a key role in the pathogenesis of MDD, while an ongoing depressive episode enhances the effect of cytokines in response to stressors and pathogens (33).

According to previous research, pro-inflammatory cytokines are assumed to play a role in the development of stress response (34) and MDD (35), both of which are major risk factors of suicidality (36). It is yet to be discovered whether different levels of the same immunological mechanism or several activated inflammatory pathways are responsible for the emergence of suicidality and the pathogenesis of MDD, however, elevated levels of immunological mediators were observed in relation to both suicidal individuals (37, 38) and patients with MDD (39, 40). These observations support the assumption of an enhancement of the immune response in the background of suicidality (41).

Altered cytokine levels may be indicative of the severity of the immunological processes as either the background or the result of suicidality and MDD, however, a more accessible way of ascertaining the extent of inflammation may be achieved by measuring laboratory parameters such as cell numbers and ratios, as they are responsible for the production of these mediators and thus the development of the immune response (42). In this current study, we aimed to examine alterations of the number of neutrophil granulocytes, lymphocytes, monocytes, platelets, WBC, NLR, MLR, PLR, MPV, ESR and RDW in MDD patients in relation to SR.

Neutrophil granulocyte count and NLR were significantly elevated in individuals with recent SA compared to patients with no history of SA. Our results support previous findings of NLR as a biomarker of SR (15, 17).

Furthermore, we found a significant increase in monocyte count and MLR in patients with recent SA compared to participants with no history of SA. Studies have previously suggested the role of MLR as an indicator of acute SR. Ucuz et al. have measured immune cell counts in blood samples taken from adolescent psychiatric patients within the first six hours after SA and compared them to those of age- and gender-matched healthy participants. They found that MLR was elevated in the former group (16), implying that an increase in MLR might be indicative of acute SR.

Moreover, WBC was elevated in patients with recent SA compared to individuals with no history of SA. An inflammatory profile with increased WBC has been linked to SR in previous research (38). Further supporting the immunological background of suicidality, Chang et al. have found that MDD patients with current suicidal ideations exhibited higher ESR values (43). Similarly, we detected an increase in ESR in participants with recent SA compared to patients with no history of SA.

As for further investigated parameters (lymphocyte and platelet count, PLR, MPV, RDW), we found no significant differences between patients with recent SA and participants with no history of SA.

Comparing high SR individuals (recent and past attempters) to intermediate SR participants (MDD patients with no history of SA), we detected a significant increase in monocyte count and MLR in the former group. Nowak et al. studied alterations of the three phenotypes of monocytes (classical, intermedier and non-classical) based on the Cluster of Differentiation (CD) molecules expressed on the surface of the cells. In patients with MDD, the number of intermedier cells and non-classical monocytes were elevated, while classical monocytes were present in lesser quantity compared to healthy controls (44). The latter monocyte group showed an increased activation state, with elevated intracellular levels of IL-6 and IL-12. It was assumed that the activation of classical monocytes led to cytokine release, which resulted in the transformation of monocytes to intermedier and finally to non-classical phenotypes (44). This is characteristic of inflammatory and autoimmune processes (45), therefore these results support the assumption of immunological mechanisms in the pathogenesis of MDD (44). Moreover, a postmortem study of suicide victims with MDD revealed increased perivascular macrophage density of the vessels in the dorsal anterior cingulate, a brain region thought to be responsible for mood disorders. Elevated levels of macrophage chemoattractant protein-1, a mediator responsible for recruiting phagocytes, have also been described (46). Furthermore, CD45 – a marker expressed in perivascular macrophages – was found to be present in greater amount compared to healthy controls. These findings imply the manifestation of inflammatory processes assumed in the background of MDD and suicidality in the form of monocyte recruitment in the brain (46).

Furthermore, there was a significant elevation in RDW and ESR in high SR patients compared to intermediate SR participants. Previous research has found elevated levels of RDW in MDD patients compared to healthy controls (47), and the increase in these inflammatory markers is in accord with the hypothesis that immunological processes may play a part in the pathogenesis of MDD. Our results infer that an elevation in ESR and RDW observed in recent and past attempters compared to patients with no history of SA might indicate a higher level of inflammation underlying suicidal vulnerability in MDD.

Although neutrophil granulocyte count, NLR and WBC were significantly elevated comparing individuals with recent SA to patients with no history of SA, we detected no significant differences regarding these parameters between the high SR and intermediate SR groups. We detected no significant elevation in the rest of the investigated laboratory values (lymphocyte and platelet count, PLR, MPV). However, all of the participants in our study were undergoing AD treatment and a subset of them were also treated with AP medication. Several studies have investigated the anti-inflammatory effect of AD therapy in MDD (18). Hannestad et al. described a decrease in the levels of proinflammatory cytokines IL-1 and IL-6 following AD treatment in MDD patients (48). According to another study, AD medication led to decreased IL-2 and increased IL-4 and TGF-β levels, the latter two being anti-inflammatory cytokines (49). Another research observed a decrease in IL-1, IFN-γ and cortisol levels in MDD patients following AD treatment. AP therapy has also been proved to have anti-inflammatory effects (50). Long-term administration of these types of pharmacotherapies may have masked changes in cell numbers, either by decreasing the severity of inflammation or preventing immunological mechanisms related to MDD or suicidality from taking place in their entirety. In a previous study on patients with MDD, elevated WBC was reduced following AD treatment, alluding to an anti-inflammatory effect of the agents independent of their AD mechanism of action (18). In a research conducted by Demircan et al., initially elevated NLR levels of drug-naive MDD patients decreased following AD treatment (47), inferring that an acute increase in these inflammatory markers may be suppressed by AD pharmacotherapy in the long term.

Therefore, we aimed to investigate the effect of these types of pharmacotherapies on the examined parameters. We found that AD treatment significantly decreased neutrophil granulocyte count and NLR, while causing no significant alterations in monocyte count and MLR. Furthermore, since a subset of patients received AP medication, we compared AP-treated patients to untreated participants and found a significant decrease in ESR in the former group. There was no significant difference regarding the number of patients treated with AP medication between the recent SA group and the no history of SA group. Although there were significantly more patients treated with AP in the past SA group compared to the no history of SA group, ESR was elevated in high SR participants (recent and past attempters) compared to intermediate SR patients (individuals with no history of SA), highlighting its reliability as a possible indicator of SR. AP treatment did not significantly influence the rest of the parameters.

Investigating diagnostic values, monocyte count and MLR had an acceptable diagnostic performance, while neutrophil granulocyte count, NLR, WBC and ESR had a limited diagnostic value.

These findings further emphasize the prognostic value of monocyte count and MLR as parameters remaining significantly elevated in not only patients with recent SA, but in all participants with high SR undergoing pharmacological treatment, therefore preserving their role as biomarkers indicating both acute and long-term SR in MDD. Considering that an increased risk of suicidality is an adverse side effect to be reckoned with especially in the first weeks of AD treatment (51), it is of great importance to have an indicator of high SR which does not solely correlate with the level of inflammation related to acute SR potentially mitigated by pharmacotherapy in the long term, but one that reliably signals the probability of an upcoming attempt independently.

In conclusion, our results are in accord with previous findings that an elevation in inflammatory markers on the one hand alludes to the contribution of immunological processes to the emergence of suicidality in MDD, and on the other hand, the investigated laboratory parameters may serve as easily accessible, cost-effective biomarkers of high SR.

Our findings imply that beyond NLR, a previously described biomarker of suicidal vulnerability, which may be significantly impacted by pharmacological treatment, MLR, a parameter exhibiting alterations which are not attenuated by the possible anti-inflammatory effects of pharmacotherapy, may therefore be a reliable indicator of not only acute but long-term SR in MDD. We hope that in the future, these biomarkers, possibly as an addition to pre-existing methods, may enable more accurate risk stratification for vulnerable patients and thus help prevent suicide from taking yet another life.

## 5 Limitations

Our research solely involved psychiatric patients diagnosed with MDD, as we aimed to detect changes of the investigated parameters related to high SR among depressed individuals as a vulnerable subset of the general population. Although suicidality is often linked to the presence of a psychiatric illness, measuring these parameters in suicide attempters without a psychiatric diagnosis may be helpful to ascertain alterations related exclusively to SR.

As the severity of MDD was not measured in this study, it would be of great use to apply rating scales to determine the correlation between illness severity, suicidality and inflammatory status more precisely.

We excluded patients attempting suicide by causing physical injury and individuals taking anti-inflammatory or immunosuppressant medication. However, recent SA was carried out by self-poisoning with benzodiazepines, which have been described to have anti-inflammatory effects (52, 53). Furthermore, other types of medication used to treat concomitant illnesses (e.g. hypertension or diabetes mellitus) may have influenced the inflammatory status of the participants. Although there is previous research inferring that AD and AP medication mitigate immunological processes, differences may be found regarding the extent of anti-inflammatory effect each subgroup of these types of pharmacotherapies might have.

Measuring biomarkers multiple times (i.e. before and at specific times after an attempt) may lead to more precise differentiation between the changes related to the emergence of suicidality and those in association with the course of illness in MDD.

We chose to measure laboratory parameters that are easily accessible, reproducible and cost-effective in a clinical setting. However, investigating changes in other inflammatory markers such as specific cytokines may lead to more precise risk assessment regarding suicidality in MDD.

## Data availability statement

The original contributions presented in the study are included in the article/supplementary material. Further inquiries can be directed to the corresponding author.

## Ethics statement

Ethical approval was not required for the study involving humans in accordance with the local legislation and institutional requirements. Written informed consent to participate in this study was not required from the participants or the participants’ legal guardians/next of kin in accordance with the national legislation and the institutional requirements.

## Author contributions

BP: Writing – original draft. MK: Writing – review & editing. DS: Writing – review & editing. TT: Writing – review & editing. AH: Writing – review & editing. TC: Writing – review & editing. DH: Writing – review & editing. NA: Writing – review & editing. EV: Writing – review & editing. MH: Writing – review & editing. PO: Writing – review & editing. VV: Writing – review & editing. TT: Writing – review & editing. RH: Writing – review & editing.
